# College Students’ Opinions About Coping Strategies for Mental Health Problems, Suicide Ideation, and Self-Harm During COVID-19

**DOI:** 10.3389/fpsyg.2022.918891

**Published:** 2022-07-06

**Authors:** Hillary Klonoff-Cohen

**Affiliations:** Department of Kinesiology and Community Health, University of Illinois Urbana-Champaign, Champaign, IL, United States

**Keywords:** coping, college students, COVID-19, mental health problems, suicide ideation, self-harm, skills training, family support

## Abstract

**Background:**

Mental health problems have emerged as a significant health complication in United States colleges during COVID-19, and as a result, they have been extensively investigated in the United States and internationally. In contrast, research on coping among the college population during the pandemic is scant. Hence, this study investigated coping strategies proposed by undergraduate students attending a Midwestern university.

**Objectives:**

The purpose of this preliminary study was to obtain college students’ feedback/opinions about coping strategies for mental health problems, suicide ideation, and self-harm during COVID-19.

**Methods:**

In December 2021, one-hundred and four undergraduate students (ages 18–22 years) completed an online survey on coping strategies using Qualtrics. Major topics included: (1) Types of coping strategies/styles expressed by students for general mental health problems, (2) Types of coping strategies for suicide ideation and self-harm behaviors, (3) Preferred platforms for receiving coping therapy, and (4) Reasons for accepting or refusing parent involvement with mental health problems.

**Results:**

The most beneficial coping strategies for mental health were ranked by college students as follows: (1) a skills training development program (30%), (2) meditation (19%), and (3) mindfulness exercises (15%), and physical education (11%). The respondents’ best coping strategies for preventing self-harm and suicide ideation/behaviors during COVID-19 were ranked as: (1) improving support from friends (32%), (2) building self-esteem (29%), and (3) addressing anger, depression, stress, and loneliness (25%). Finally, a total of 50% of participants felt that parents should be involved in college student interventions. Students stated that the most important type of support that they received from their parents were: (1) emotional support (31%), (2) direction and/or assistance with solutions (27%), and problem-solving (16%).

**Conclusion:**

This study identified potential avenues which could be implemented into action during future outbreaks. Specifically, employing interventions that: (i) train undergraduate students to employ more effective skills training coping strategies or practicing mindfulness or meditation; (ii) integrate mental health, suicide, and self-harm prevention into the curriculum; (iii) offer more in-person campus services targeted toward the psychological and emotional effects of a pandemic, and (iv) involve support persons (e.g., family) in students’ lives to enhance their well-being during and after COVID-19.

## Introduction

The unanticipated and traumatic effects of COVID-19 and quarantining since January 2020 have negatively impacted the mental health of undergraduate college students. Researchers have primarily evaluated social stress and academic stress (Vidal Bustamante et al., 2022), anxiety (Brooks et al., 2020; Kochuvilayil et al., 2021; Bountress et al., 2022; Kaur et al., 2022; Song et al., 2022; Tshering and Dema, 2022), anger (Brooks et al., 2020), and depression (Brooks et al., 2020; Bountress et al., 2022; Tshering and Dema, 2022) in this vulnerable population. In addition, a plethora of other reported outcomes during COVID-19 included loneliness (Xiang et al., 2020; Kaur et al., 2022), isolation (Hasratian et al., 2021; Kaur et al., 2022), sleep disturbance/difficulty sleeping or insomnia (Kochuvilayil et al., 2021; Zhang et al., 2021; Song et al., 2022), fatigue (Mosleh et al., 2022), burnout (Kaggwa et al., 2021), PTSD (Brooks et al., 2020; Bountress et al., 2022), future uncertainty (Miconi et al., 2022), witnessing death in relatives (Hasratian et al., 2021), relocation/displacement (Hasratian et al., 2021), alcohol (Bountress et al., 2022), e-cigarette (Merianos et al., 2022), and cannabis use (Merianos et al., 2022; Merrill et al., 2022), financial insecurities, loss or stressors of income (Hasratian et al., 2021), unhealthy eating behaviors (Kochuvilayil et al., 2021), academic concerns (Kochuvilayil et al., 2021; Vidal Bustamante et al., 2022), contagion (i.e., fear of contracting the disease; Wheaton et al., 2021), mobile phone addiction (Jiang et al., 2022; Peng et al., 2022), relationship problems (Gallegos et al., 2021; Herbenick et al., 2022), sexual activity problems (Ellakany et al., 2022; Herbenick et al., 2022), increased screen time (Ellakany et al., 2022), suicidal behavior (López Steinmetz et al., 2021; Bountress et al., 2022; Rahman et al., 2022), self-harm (Kim et al., 2021), and fear of death (Xiang et al., 2020).

The overwhelming majority of studies on this population have focused on the symptomology of mental illness. Prevention of COVID-related psychological effects through coping strategies in college students is profoundly absent from the literature.

Hence, our study sought to address this gap by obtaining undergraduate University of Illinois students’ opinions on the most appealing/beneficial coping strategies that would empower college students during the ongoing pandemic as well as determine which coping strategies would lessen or prevent self-harm and suicide ideation/behaviors. Finally, students were queried about what type of support parents provided to students while attending college. We intentionally did not inquire about students’ mental health problems, treatments, and suicide and self-harm histories. Rather, we purposely concentrated on students’ perceptions of how to best tackle mental health issues in a college population.

There are three types of coping strategies: problem-focused, emotion-focused, and avoidance-focused situations described in the literature (Baqutayan, 2015). The most widely reported problem-focused coping strategies are active coping (e.g., problem understanding and solving) and seeking social support for instrumental reasons (e.g., asking others for help and developing social support; Chaabane et al., 2021). Positive reinforcement and growth (e.g., staying optimistic and wishful thinking) and turning to religion, are the most widely used emotion-focused stress coping strategies (Chaabane et al., 2021). In contrast, the most commonly used types of avoidance or dysfunctional coping strategies are mental disengagement (e.g., transference, becoming involved in other activities) and behavioral disengagement (e.g., avoidance, social withdrawal; Chaabane et al., 2021). In our study, coping choices centered around active coping, seeking social support, and mental disengagement.

## Methods

### Participants and Procedure

An online survey was administered through Qualtrics to Community Health majors enrolled in a Public Health (PH) Research Methods course at the University of Illinois Urbana-Champaign in Fall 2021. Study participants were compensated for their time with five extra credit points. A total of 104 students in the PH methods course completed the survey, reflecting a 100% response rate. Students were eligible if there were 18 years of age or older and enrolled in the above-mentioned PH course.

All participants received a link to Qualtrics that included an electronic informed consent document explaining the study, the brief survey, and a separate form used to collect information for assigning bonus participation points. Participants concluded the survey online and the average time to completion was about 5–8 min. This study involved human participants and was reviewed and approved by the University of Illinois Institutional Review Board. The participants provided their written informed consent to participate in this study.

### Measures

A newly designed 9-item survey instrument was designed to assess: (1) Types of coping strategies/styles expressed by students for general mental health problems, (2) Types of coping strategies/styles expressed by students for suicide ideation, and self-harm behaviors, (3) Preferred platforms for receiving coping therapy, and (4) Reasons for accepting or refusing parent involvement with mental health problems. The initial approach to designing this survey was to focus on coping by promoting strengths and protective factors rather than on targeting deficits (Houston et al., 2017). Students were asked to rank (from highest to the lowest order of preference) potential coping strategies to: (i) promote good mental health, and (ii) prevent suicide ideation and self-harm behaviors. They were also provided with an “other” category to disclose additional strategies of their choosing.

### Statistical Analysis

All analyses were descriptive in nature and were performed using Qualtrics.

## Results

The majority of students in this study were 19 or 20 years of age (76%). Half of the students were enrolled as juniors, while the rest were sophomores (37%), or seniors (13%). Eighty percent of the students identified themselves as female. Forty percent of participants were white, 24% were black, 21% were Asian, 15% were other (Latino/a, Hispanic, or interracial), and 2% were American Indian or Alaska Native (Table 1).

**TABLE 1:** Demographics of the undergraduate college students.

Field	*N* Students	(%)
Gender		
Female	19	20%
Male	76	80%
Age		
19	30	32%
20	42	44%
21	18	19%
22	5	5%
Race		
White	41	39%
Black/African American	24	23%
American Indian/Alaska Native	2	2%
Asian	21	20%
Native Hawaiian/Pacific Islander	2	2%
Other	15	14%
Year		
Sophomore	35	37%
Junior	48	51%
Senior	12	13%

The most beneficial coping strategies for mental health were ranked by college students as follows: (1) a coping skills development program (30%), (2) meditation (19%), (3) mindfulness exercises (15%), and physical education (11%; Table 2).

**TABLE 2:** Ranking of coping strategies for mental health by college students.

Coping strategies*	Ranked as 1st choice		Ranked as 2nd choice		Ranked as 3rd choice	
	*N*	%	*N*	%	*N*	%
Coping development program to enhance mental health	25	29%	7	8%	10	12%
Mindfulness exercises	15	17%	19	22%	13	15%
Meditation	19	22%	16	19%	13	15%
Mindfulness based Art therapy	9	10%	6	7%	11	13%
Physical education (in person or web based)	11	13%	12	14%	12	14%
Peer support programs	9	10%	11	13%	12	14%
Use of social media for relaxation technique	7	9%	9	11%	7	9%

* “Other” category was not presented because none of the students ranked it in their top three choices.

The respondents’ best coping strategies for preventing self-harm and suicide ideation/behaviors during COVID-19 were ranked in this way: (1) improving support from friends (32%), (2) building self-esteem (29%), (3) addressing anger, depression, stress, and loneliness (25%), (4) improving support (building bridges) with family (19%), (5) managing substance abuse (10%), (6) improving or developing resilience (10%), and (7) enhancing time management and goal-oriented behaviors (5%; Table 3).

**TABLE 3:** Ranking of coping strategies for suicide ideation and self-harm behaviors.

Coping strategies*	Ranked as 1st choice		Ranked as 2nd choice		Ranked as 3rd choice	
	*N*	%	*N*	%	*N*	%
Improving support from friends	28	32%	11	13%	8	9%
Improving support (Building bridges) with family	19	22%	13	15%	15	17%
Building self-esteem	25	28%	17	19%	13	15%
Addressing anger, depression, stress, and loneliness	22	25%	12	14%	15	17%
Managing substance abuse	9	10%	5	6%	10	11%
Enhancing time-management and goal-oriented behaviors	5	6%	13	15%	4	5%
Improving or developing resilience	9	10%	7	8%	7	8%
Working at a job (Volunteer or paid)	7	8%	3	3%	3	3%

* “Other” category was not presented because none of the students ranked it in their top three choices.

The platforms that were deemed most beneficial by students in order to help them deal with mental health issues were: (1) visiting the campus Counseling Center or other mental health services in- person (68%), (2) going to resident advisor-led training sessions in-person (19%), and (3) attending resident advisor-led training sessions online (13%).

Finally, a total of 50% of participants felt their parents should be involved in college coping interventions for mental health while 35% were unsure about their parents’ participation. The remaining 15% of students reported that parents should not be involved in college interventions that employed coping strategies.

Students stated that the most important type of support that they received from their parents was: (1) emotional support (31%), (2) direction and/or assistance with solutions (27%), (3) problem-solving (16%), or (4) a calming effect (13%; Table 4).

**TABLE 4:** Type of support offered by parents to undergraduate college students.

Type of support:	*N*	%
Problem solving	14	16
Emotional support	28	32
Calming effect	11	13
Providing direction and/or assisting with solutions	24	27
I do not currently ask for and/or receive support from my parents/family	11	13

## Discussion

Our study revealed that during COVID-19, skills training, meditation, and mindfulness were the preferred methods chosen by undergraduate college students to cope with mental health problems. Additionally, in the areas of self-harm and suicide ideation, students perceived support from friends and family as most beneficial.

### Skills Training

Skills training development programs protect mental health and prevent suicide for teens and young adults in the United States (JED Foundation, 2021). There were no United States studies that reported the impact of skills training and coping during the pandemic in college students. However, in a study of 386 Nigerian undergraduate students (with a 21% response rate), students coped with the lockdown and the cessation of academic activities during COVID-19 by engaging in online skills-acquisition building activities (15.3%), using social media (17.9%), and watching television or videos (11.1%; Ojewale, 2021). Face-to-face skills-building activities were not evaluated in this study (Ojewale, 2021).

### Mindfulness and Meditation

An online intervention was used to test (Dorais and Gutierrez, 2021) the effectiveness of a 4-week centering meditation treatment, which proved to be successful in improving levels of stress and trait mindfulness in a college population (Dorais and Gutierrez, 2021). A second randomized clinical trial assessed the effectiveness of an 8-week, web-based mindfulness and cognitive behavioral therapy program in reducing symptoms of depression, anxiety, and stress (primary outcomes) and increasing mindfulness (secondary outcome) in 160 undergraduate students at a Canadian university. Using video-based modules, peer-to-peer discussions, and anonymous group-based video discussions, there were significantly reduced depression and anxiety symptoms but no effects on perceived stress (El Morr et al., 2020). The Koru mindfulness 4-week curriculum (embedded within a college course) intervention increased state mindfulness, decreased stress, and improved sleep during the pandemic in 34 undergraduate students compared to students (*N* = 35) enrolled in a different course (Smit and Stavrulaki, 2021). A pilot trial suggested that both mindfulness and social support delivered *via* mobile Health, showed promise in reducing distress among 114 young college adults in quarantine in China, with mindfulness being particularly effective in addressing anxiety (Sun et al., 2022). Last, in 99 college students ranging in age from 18 to 29 years, emotional intelligence and mindfulness training using the Ajivar app during COVID-19 resulted in improvements in anxiety, depression, and emotional intelligence (Sturgill et al., 2021).

### Face to Face vs. Online Mental Health Care

In contrast to the above mentioned five studies, our study determined that students greatly preferred face-to-face mental health care rather than online platforms in order to receive coping strategies. This could be a result of our study being performed 2 years into the pandemic when isolation and loneliness were very prevalent. In one recent small Italian study (*n* = 34), the online counseling intervention was almost as effective as the face-to-face counseling intervention with respect to psychological distress; however, face-to-face counseling was superior to online counseling with regard to university students’ life satisfaction before and during COVID (Ierardi et al., 2022). Since life satisfaction is associated with better physical health, higher performance, and stronger social relationships (Tsaousides, 2018), this is a meaningful finding when considering university students’ quality of life. In the future, more in-person mental health and coping interventions should be explored within college populations.

### Social Support

In our study, both parent and friend involvement were identified as coping strategies for suicide ideation and self-harm within our college population during the COVID-19 epidemic. Within the literature, only one study involved friends, proposing the fitness buddy program model as an innovative and cost-efficient strategy to support college students’ psychological well-being and long-term success during COVID (Kirby et al., 2022). With respect to family support, one of the main coping activities employed by 381 University Jordanian students, ages 18–38 years, consisted of more engagement with family (53%); albeit, the most common reported coping strategy was spending more time on social media platforms (71%; Al-Tammemi et al., 2020).

Another study reported that healthy family function may alleviate general anxiety disorder and anxiety of college students during the COVID-19 pandemic (Yang et al., 2021). A cross-sectional study in 2020 consisting of 558 undergraduate students from seven geographical regions across China reported that a large percentage of college students adopted passive coping strategies such as smoking and drinking, which were detrimental to their mental health (Huang et al., 2021). However, family support was very important for protecting against anxiety, depression, and stress (Huang et al., 2021).

### Limitations

Skill building, meditation, mindfulness, friends’ support, addressing self-esteem, managing anger, depression, stress, and loneliness, and involving parents surfaced as top-ranking coping strategies in our study. Nevertheless, several limitations should be acknowledged. This was a cross-sectional design so causal relationships between coping strategies and decreased mental health problems could not be inferred. All coping strategies were inquired through self-report and no alternative assessments were used. Students were not queried about their own mental health problems or whether they received help and support for them; rather, they only provided their beliefs/opinions about coping strategies. Thus, mental health diagnoses were not obtained or verified. Data were only collected from a single Midwestern college among students in a health-related major and convenience sampling was used to recruit participants; therefore, generalization of the findings is limited. This study contained a small sample size which did not allow for the analysis of predictor variables and examination of interaction effects. The cohort may not be fully representative of a student population based on the narrow age range and majority of the sample being female. It is possible that students may be more psychologically minded and aware of the types of beneficial help and support for emotionally related situations in light of them being “majors enrolled in a PH Research Methods course.” Additionally, the 100% response rate could reflect that students were motivated to help in the field of understanding mental health issues or alternatively, their motivation could be a result of only being interested in receiving five extra credit points.

### Future Recommendations

The importance of what students can do for themselves, as well as what their friends and family can do to help to support and positively influence them, are important findings not just in relation to COVID-19 but also in the world post-COVID. Based on the results of this descriptive study, examples of specific university actions that may warrant further evaluation using randomized clinical trials Apr include: (1) engaging and educating parents about how to best support their loved ones on campus during and after any pandemic, (2) determining how to expand platforms to support face-to-face student mental health during crises, and (3) offering self-care student activities on campuses such as coping skills development/building, mindfulness, and meditation classes.

## Data Availability Statement

The raw data supporting the conclusions of this article will be made available by the authors, without undue reservation.

## Ethics Statement

This study involved human participants and was reviewed and approved by the University of Illinois Institutional Review Board. The participants provided their written informed consent to participate in this study.

## Author Contributions

The author conceptualized the research and methodology, obtained human subjects approval, participated in draft writing and manuscript preparation, administered the surveys online, and approved the final manuscript.

## Conflict of Interest

The author declares that the research was conducted in the absence of any commercial or financial relationships that could be construed as a potential conflict of interest.

## Publisher’s Note

All claims expressed in this article are solely those of the authors and do not necessarily represent those of their affiliated organizations, or those of the publisher, the editors and the reviewers. Any product that may be evaluated in this article, or claim that may be made by its manufacturer, is not guaranteed or endorsed by the publisher.
